# Evaluation of the true lateral fluoroscopic projection for the relation of the S1 recess/foramen to safe corridors in transiliac-transsacral screw placement in human cadaveric pelves

**DOI:** 10.1007/s00590-024-04157-5

**Published:** 2024-11-28

**Authors:** Fabian Cedric Aregger, Jan Gewiess, Christoph Emanuel Albers, Moritz Caspar Deml, Samuel Schaible, Sven Hoppe, Christian Tinner

**Affiliations:** 1 Department of Orthopaedic Surgery and Traumatology, Inselspital, Bern University Hospital, University of Bern, 3010 Bern, Switzerland; 2 Wirbelsäulenmedizin Bern AG, Bern, Switzerland

**Keywords:** Sacrum, Osteoporosis, Fracture, Screw osteosynthesis, Cadaveric

## Abstract

**Introduction:**

Percutaneous screw fixation is a widely used treatment for posterior pelvic ring injuries. Transiliac-transsacral screw fixation has demonstrated superior biomechanical properties over bilateral sacroiliac screws, particularly in the minimally displaced bilateral sacral fractures. Screw placement under fluoroscopic control is still common, while CT navigation is gaining popularity. However, the accurate placement of screws within a safe zone is essential to avoid neurovascular complications.

**Methods:**

An anatomical study using human cadaveric pelves was conducted to assess radiological landmarks and determine a safe zone in relation to the S1 recess/foramen for transiliac-transsacral screw placement.

**Results:**

Fourteen pelves were evaluated. Ten pelves were classified as having a satisfactory corridor for screw placement, while four were deemed to have an impossible or high-risk corridor. A safe zone was defined based on the diagonal bisector of the S1 vertebral body, ICD and anterior cortex.

**Discussion:**

The study findings suggest that lateral fluoroscopic projection can determine a safe entry point for screw placement. Understanding the anatomy and landmarks on lateral fluoroscopic images is crucial for successful screw placement and avoiding complications.

**Conclusion:**

The S1 body diagonal is consistently located anterior to the S1 recess in lateral fluoroscopic projections, providing a potential safe corridor for transiliac-transsacral screw placement at the S1 level in nondysmorphic pelves. Further research is needed to confirm these findings with CT imaging and evaluate the technical feasibility of screw placement.

## Introduction

Percutaneous screw fixation is an effective treatment for unstable posterior pelvic ring injuries [1–3]. Transiliac-transsacral screw fixation has shown superior biomechanical characteristics compared to bilateral sacroiliac screws, especially in minimally displaced bilateral sacral fractures or sacroiliac joint disruptions [4]. However, the morphology of the sacrum heavily influences the presence of a suitable transsacral corridor indicated by a sufficiently large safe zone that can accommodate 6.5–7.3 mm transiliac-transsacral screws. Screw malpositioning outside of the safe zone carries a risk of neurovascular compromise, with iatrogenic neurologic complications reported in up to 25% of patients in retrospective studies [2, 5–9].

Thus, it is crucial to not only assess the availability and size of transsacral corridors on CT scans before the procedure, but also to assess intraoperatively for safe screw trajectory [10]. While CT navigation is becoming more popular, the current standard technique still involves freehand screw insertion under fluoroscopic control [5, 11]. Therefore, it is essential to identify important anatomical landmarks intraoperatively using fluoroscopic views [12]. Key landmarks for this assessment include the sacral foramina on the outlet projection, the spinal canal and S1 body on the inlet projection, and the iliac cortical densities on a true lateral projection (which define the anterosuperior boundary of the ‘safe zone’ for screw insertion). However, in addition to anatomical variations in sacral morphology and lumbosacral lordosis, the complex shape of the posterior pelvis along with bone overlap, obesity, poor bone quality, and bowel gas can limit the detection of the S1 recess/foramen on inlet/outlet projections [13]. The iliac cortical density line helps not only in the evaluation of orientation and alignment of the pelvis in radiographic imaging but also defines a bony demarcation within the true pelvis. As an essential anatomical landmark, it indicates the radiographic-anatomical proximity or the separation of the implant from the L5 nerve root. Additionally, the trajectory of the S1 neural recess progressing to the ventral foramen is a 'structure at risk'. Unlike the iliac cortical density lines serving as radiological landmarks for the location of the L5 nerve roots, a radiologic demarcation of the S1 lateral recess and foramen is absent in the lateral projection.

To the best of our knowledge, the existing literature consistently lacks a detailed depiction of the trajectory of the S1 nerve root within the recess preceding its exit from the foramen on the lateral fluoroscopic projection. In an anatomical study using human cadaveric pelves, we aim to assess the accuracy of using true lateral fluoroscopy alone to determine the dimensions of the S1 recess/foramen and identify a safe zone/entry point for transiliac-transsacral screw placement with respect to the S1 recess/foramen.

## Methods

### Hypothesis

We hypothesized that a strictly lateral image could be used to assess the feasibility and identify an entry point for transiliac screw placement in relation to the S1 recess by utilizing common anatomical landmarks.

Our primary objective was to visualize the S1 recess/foramen utilizing the true lateral fluoroscopic projection only.

The secondary objective was to define a corridor transiliac-transsacral screw placement which is reliably outside the S1-recess utilizing the true lateral fluoroscopic projection only.

### Material & methods

14 human cadaveric dried osseous pelvic specimens without macroscopic anomalities from our affiliated anatomical institute were procured. Unstable parts were additionally stabilized with transosseous wires.

The pelves were placed supine on operating tables. For image intensification, a C-arm (Ziehm Vision RFD 3D) was utilized.

#### Lateral projection

At our department an image of the pelvis is considered as true lateral if the iliac cortical density adjacent to the sacroiliac joint parallels the sacral alar slope. In our setting, the projection was centred at the S1 level, displaying the iliac cortical density line bilaterally in congruence. Rotation was assessed based on facet joints and sciatic foramina.

#### Bony demarcation

The iliac cortical-density lines and the transition between the S1 and S2 segments were defined as radiological anatomical landmarks for intraoperative orientation and determination of the implant positioning.

**Fig. 1:**
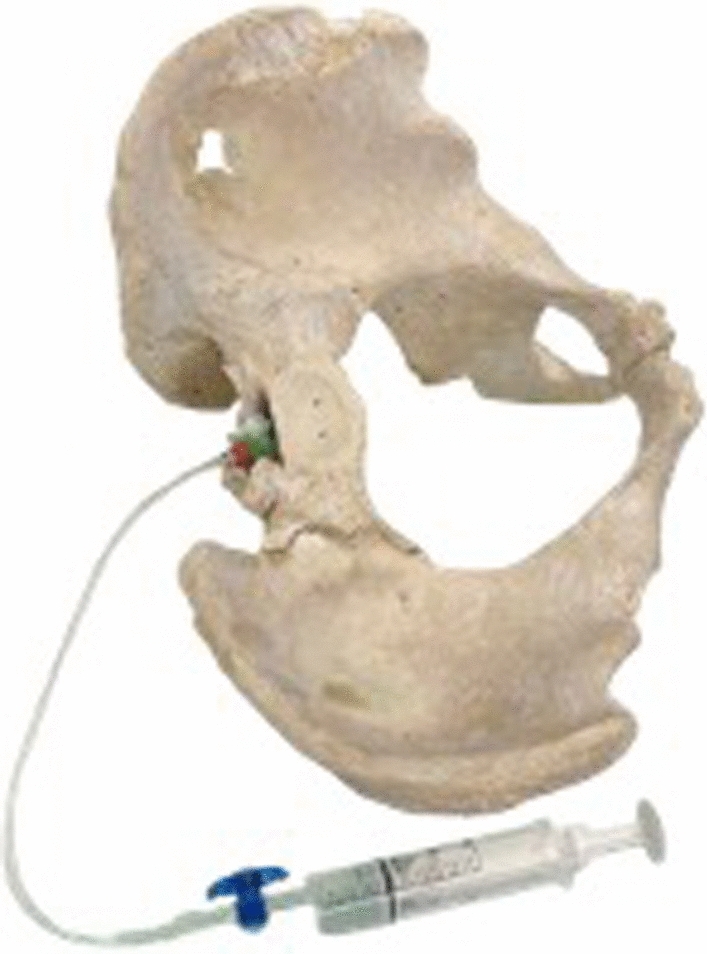
Setup: human cadaveric pelvis on operating table, inserted water balloon connected through a line and a 3-way stopcock to the contrast medium filled syringe

**Fig. 2:**
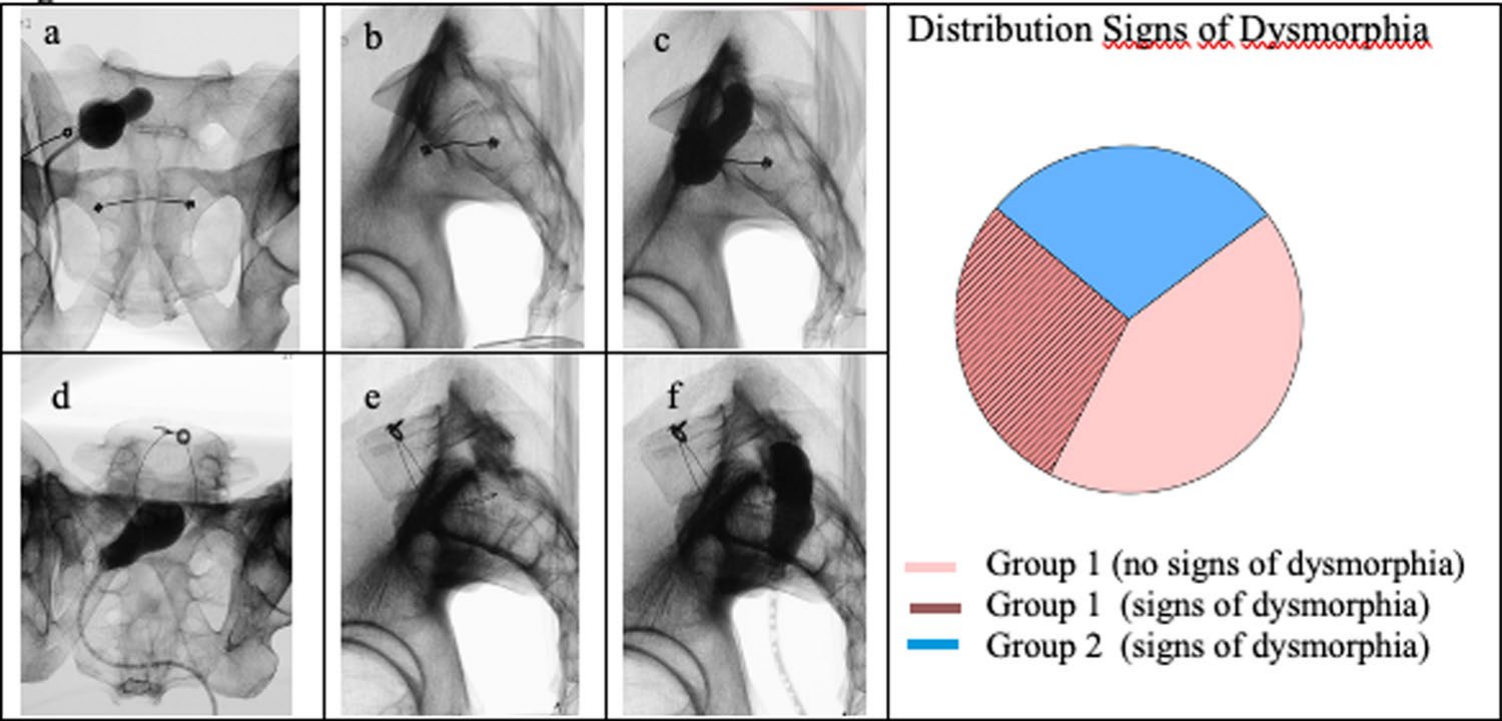
Distribution signs of dysmorphia. **a** Pelvis with residual disc between S1/2, alar mammillary process, abnormally shaped upper sacral ventral neuroforamen, abnormally shaped upper sacral ala. In the lateral view **b** close relation of the ICD and the bisector. **c** Image with contrast-filled balloon, the proximity to the S1 recess/foramen becomes apparent. **d** Pelvis without signs of dysmorphia in outlet view. **e** Large area between ICD and bisector. **f** Large area between contrast-filled-balloon and ICD

**Fig. 3:**
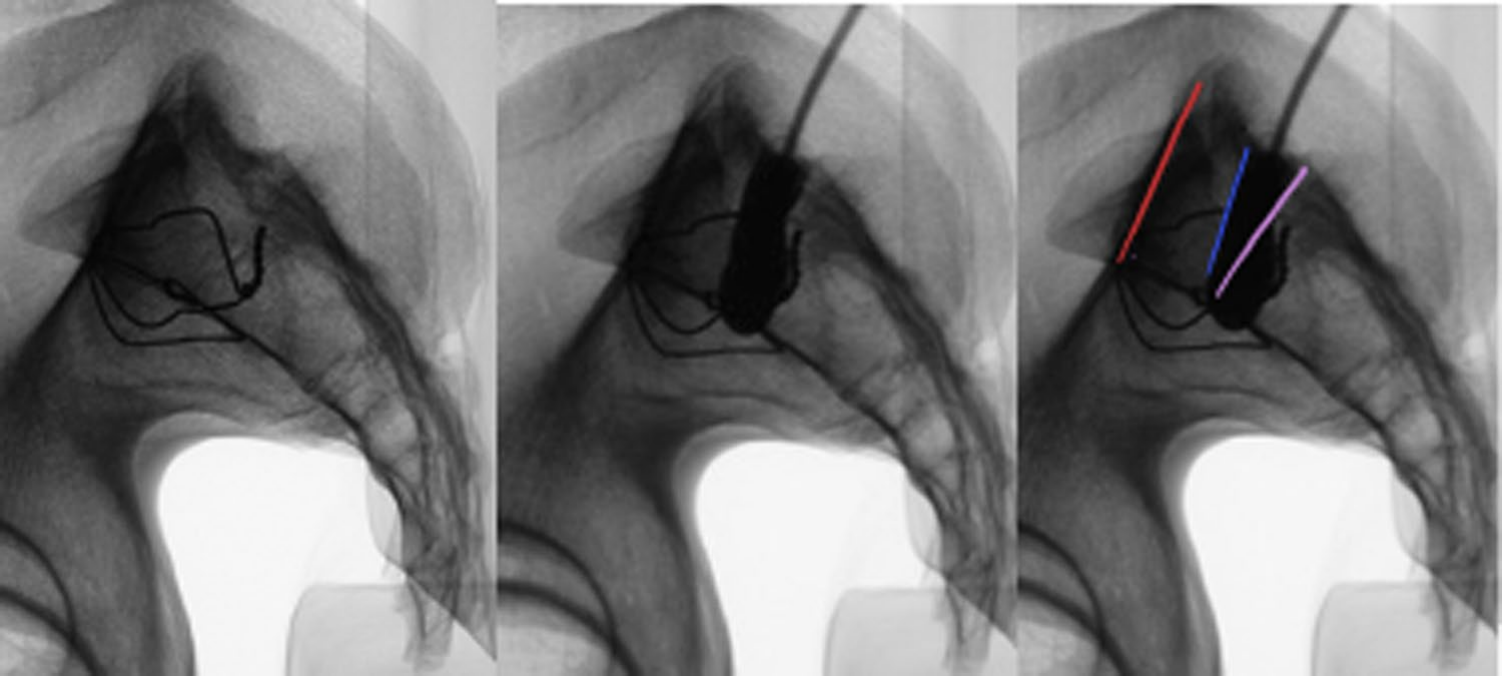
A true lateral fluoroscopic projection of the sacrum is depicted. **a** Plain projection; **b** S1 recess/foramen contrasted by the contrast medium; **c** marked iliac cortical density line (red line) and S1 cranial margin of recess/foramen (blue line), S1/S2 disc space (pink line),

To visualize the recess and foramen, a standard water balloon (5.6 cm length) was introduced into the S1-foramen and filled with contrast medium (Iopamiro, 4-8 ml) (Figs. 1, 2). The backflow from the balloon was blocked with a 3-way stopcock, and subsequently, a true lateral image was taken (Fig. 3). As the filled balloon extends along the foramen all the way to the spinal canal, the entire course of the canal surrounding the pedicle could be traced in the lateral projection.

#### Safe zone

The lateral radiographs were evaluated for their osseous corridor for transiliacal S1 screw placement by two spine surgeons. The corridor was assessed based on the lateral triangle, serving as an indicator for corridor width, according to Mendel [14]. Pelves were classified into two groups. In cases where a lateral triangle type A or B according to Mendel was present, it was considered to be sufficient for a transiliacal S1 screw placement. The first group comprised pelves that were independently evaluated by the surgeons as having a feasible corridor for a transiliacal S1 screw. The second group was considered to have an 'impossible' or 'high-risk' corridor. Additionally, the radiological signs of dysmorphism (according to Routt) were evaluated for each of the pelves. These hallmarks of sacral dysmorphism include: Colinear upper sacrum and iliac crests, residual disc between upper two sacral vertebra, alar mammillary processes on outlet images, abnormally shaped upper sacral “ala”, accentuated alar slope, “tongue-in-grove” on CT scan, ICD not coplanar with alar slope, recessed ala relative to ICD.

**Fig. 4:**
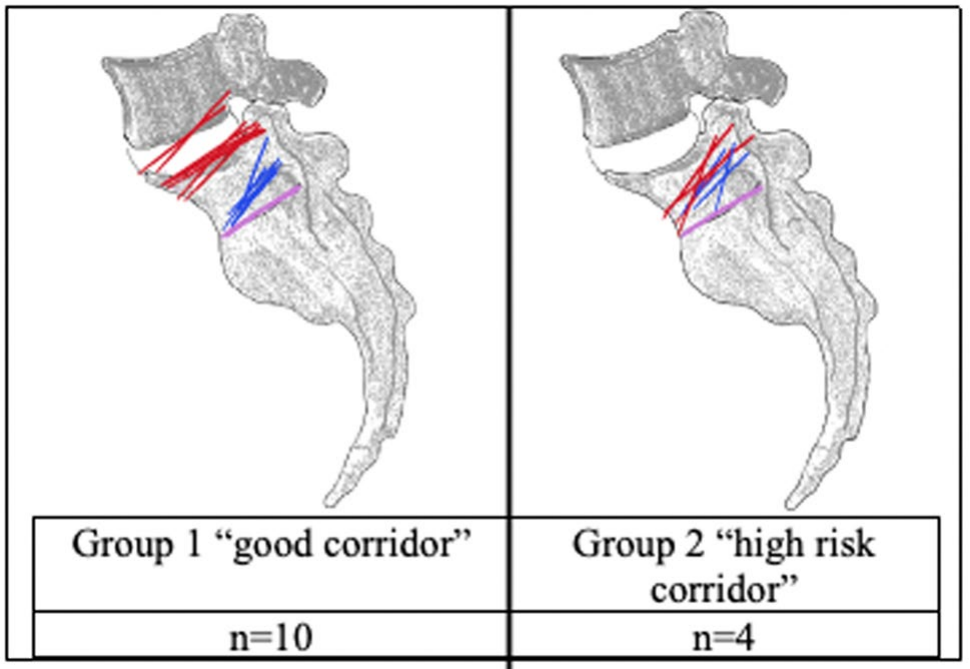
Superimposed S1 vertebrae with schematically documented structures: iliac cortical density line (red lines), S1/S2 disc space (pink line), cranial margin of S1 recess/foramen (blue line)

Subsequently, the lateral images with applicated contrast medium were schematically documented and scaled. These documents included the cortical boundary of the S1 vertebral body, the iliac cortical density line, and the S1 foramen contrasted by the contrast medium (Fig. 3). This facilitated a more straightforward assessment by superimposing all S1 vertebrae within a group (Fig. 4). Through this process, the named and schematically documented structures within a group were evaluated for notable commonalities (Fig. 4).

## Statistical analysis

### Results

A total of 14 pelves were evaluated.

#### Lateral triangle/corridor

In order to evaluate whether our described landmarks marking the S1 recess/foramen differ between dysmorphic and non-dysmorphic pelves, we stratified our collective into two groups. According to Mendel's classification, 10 (71%) out of 14 pelves presented a mean triangle ratio of 5.57 and could therefore be categorized as Mendel Type A with a satisfactory corridor and were therefore assigned to the group 1 (Table 1).The remaining four pelves presented a Type C lateral triangle (mean triangle ratio 1.32) and were therefore assigned to the second group.

**Table 1:** Division of all the pelves into 2 groups according to Mendel

	Group 1 (n = 10)	Group 2 (n = 4)
Mendel lateral triangle ratio (mean)	5.57 (3–9.3)	1.32 (1.2–1.75)
Signs of sacral dysmorphia	4 (40%)	4 (100%)

#### Group 1

In the collective of Group 1, the iliac cortical density line consistently traversed the upper half of the vertebral body in true lateral projection. The channel extending towards the S1 foramen ran below the diagonal bisecting the vertebral body in all 10 pelves.

#### Group 2

In contrast to Group 1, the iliac cortical density line in the four pelves of Group 2 traverses the lower half of the lateral projection of the S1 vertebra. Consistent with the first group, the contrasted bony boundary of the canal to the S1 foramen also runs below the diagonal that bisects the vertebral body in Group 2.

Each of these four pelves showed at least four signs of sacral dysmorphia. All four displayed an accentuated alar slope as well as an abnormally shaped ala. Three showed additionally an abnormally shaped upper sacral ventral foramen on the outlet image, alar mammillary process, and a residual disc at S1/2.

#### Safe zone

Based on the findings of both groups three assertions could be made for pelves with a sufficient corridor:

- The contrast-enhanced recess/canal of the S1 root was under 45° (in relation to the endplate) throughout the entire cohort, and it never surpassed the diagonal bisector of the vertebral body.
- The ventral S1/2 foramen was located at the height of the S1/2 segment.

**Fig. 5:**
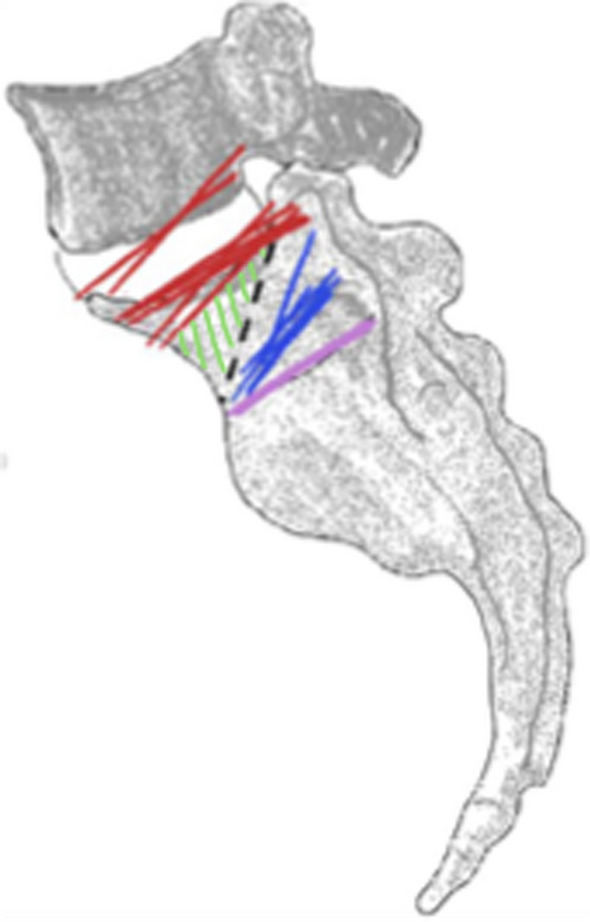
Definition of a safe zone (green) which is delineated by the diagonal of the S1 body, ICD and anterior cortex. Illustrated on sacrum with all superimposed structures of group 1

Thus, for non-dysmorphic pelves a safe zone (as shown in Fig. 5) can be defined, which is delineated by the diagonal bisector of the lateral projection of the vertebral body, the anterior cortex and the ICD.

## Discussion

In this anatomical study using human cadaveric pelves, we aimed to assess radiologic landmarks with respect to the S1 recess/foramen in lateral fluoroscopy to determine a potentially safe region for transiliac-transsacral screw placement. Our findings demonstrate that landmarks to define the region for potential screw placement can consistently be identified on non-dysmorphic pelves. Furthermore, we could demonstrate that the diagonal bisector (posterosuperior to anteroinferior) is reliably anterior to the S1 recess/foramen. Consequentially, the bisector together with the iliac cortical density and anterior cortical lines can be used intraoperatively to reliably determine which pelves are suitable for transiliac-transsacral screw placement. Previous studies primarily have focused on different methods, such as 3D statistical models of CT scans or landmark projections on standard Matta projections, to identify safe transverse corridors [14–18]. However, these studies did not specifically address the location of the S1 recess/foramen on lateral fluoroscopic images.

Transiliac-transsacral screw placement is increasingly used in the treatment of sacral fragility fractures [15]. Compared to sacroiliac screw fixation, transiliac-transsacral screw fixation offers potential benefits such as better distribution of craniocaudal forces along its longer length, increased screw thread purchase in the opposite iliac cortex, and greater compressive forces on the fracture and/or sacroiliac joint [19, 20].

Successful transiliac-transsacral screw fixation relies on the presence of a transsacral corridor with sufficient dimensions. The presence of a transverse corridor for transsacral screw placement is largely influenced by the sacral anatomy and is typically assessed using lateral and Matta projections, as well as CT scans. The safe corridor is limited by the vestibule (isthmus) representing the narrowest passage that the screw has to cross to reach the S1 body [21]. Its boundaries are the sacral canal, the sacral foramen S1, and the sacral cortex. In a CT-based biomorphometric analysis of 280 pelves conducted by Gras et al., a transsacral S1 corridor was identified in 89% of cases, with an average maximum cylindrical diameter of 13 mm [16]. Similarly, a transverse corridor for S2 was found in 279 out of 280 pelves, with an average maximum cylindrical diameter of 12 mm. It was observed that females were more likely to exhibit sacral dysmorphism compared to males, and the dimensions of the transsacral S1 and S2 corridors were significantly smaller in females. This phenomenon of a relatively wider corridor in males compared to females has also been documented by Mendel et al. [14]. Sacra that lack a sufficiently sized S1 or S2 transsacral corridor are referred to as ‘minority type’, while those lacking a sufficiently sized S1 transsacral corridor are referred to as ‘dysmorphic’[22]. ‘Pelvic dysmorphism’ has been reported in the literature to occur in 8% to 53% of patients and was observed in 29% of the pelves in our study [2, 14, 21–26]. Signs of sacral dysmorphism include the sacrum not being recessed within the pelvis, presence of mammillary processes cranial to the sacral ala, dysmorphic S1 foramen, and residual disc space at S1/S2 (outlet view), anterior cortical indentation of the anterior S1 cortex (inlet view), acute sacral slope (lateral view), and extensive interdigitation of the sacroiliac joint and a ‘tongue-in-groove’ sign (CT scan) [12]. Additionally, Kaiser et al. developed a "sacral dysmorphism score", which is calculated as the sum of the coronal angle of the 10 mm corridor in S1 plus twice the axial angle of the 10 mm corridor in S1 [22]. Pelves with scores above 70 indicate the absence of a transsacral S1 corridor. Even though a transitional anomaly was only observed in a ‘dysmorphic sacrum’ in our study, lumbosacral variations such as Castellvi types IIb and IV are not considered to be the anatomical basis for ‘sacral dysmorphism’ [27].

The majority of studies assessing the corridor are based on computed tomographic analysis. While CT evaluation is often the standard in preoperative analysis intraoperative 3d-imaging or even navigated screw placement is still reserved for a minority as it is resource-intensive. This leads to a desire for an assessment of morphology in a projection that is simple to reproduce.

The ratio of the S1 height to the S1 width from the intersecting iliac cortical density line, known as Mendel's ‘lateral sacral triangle’, has been proposed as a reliable substitute for the vestibule height [17]. The height of the vestibule is a critical factor in determining the safety of transsacral screw insertion and is known to be reduced in sacral dysmorphism [14]. Mendel et al. reported that a lateral sacral triangle ratio greater than 1.5 indicates a safe corridor for screw placement. To achieve safe transiliac-transsacral S1 screw placement within the ipsi- and contralateral vestibules, it is important to consider relevant anatomical landmarks [4]. These landmarks include the anterior boundary of the S1 ala, the lateral sacral alar slope (iliac cortical density), the height and width of the vestibule, and the height and width of the S1 body. It is consistent with our observation that all pelves assigned to the second group showed signs of sacral dysmorphism.

While above mentioned are not always easily assessed on fluoroscopic images, the height and width of the S1 vertebra can be accurately measured.

The true lateral projection used in our study is characterized by the convergence of the ipsi- and contralateral iliac cortical density lines near the sacroiliac joint. Initially, this line was believed to represent the region between the sacroiliac joint and the lateral S1 body. However, it is now understood that this line may actually represent the general area surrounding the joint, as it is formed by the uneven combination of cortical and cancellous bone [4]. Therefore, it should be considered as an approximation of the alar cortex. The iliac cortical density is typically located inferior and posterior to the sacral ala, serving as a reference for the anterior boundary of the ‘safe zone’ [29]. Consequently, when inserting screws, it is important to start inferior and posterior to this line. A commonly used alternative landmark is the greater sciatic notch, which, like the PSIS and acetabulum, when overlapping, ensure accurate assessment of rotation [28].

While these landmarks are typically evaluated on true lateral fluoroscopic projections, the superior bony surface of the S1 foramen is usually assessed on the outlet view. However, in a clinical setting, accurately identifying all these landmarks on lateral fluoroscopy can be challenging due to the dense sacral bone, surrounding soft tissue and bowel gas, as well as difficulties in distinguishing the ipsi- from the contralateral side. Even after identifying the lateral entry point, it remains desirable to establish a relationship with critical anatomical structures to ensure safe and accurate placement.

Our study demonstrated that the S1 recess/foramen does not exceed the diagonal line from the posterosuperior to anteroinferior aspect of the S1 body. The defined safe zone allows defining an entry point, while also offering an additional reference for verifying implant positioning in the true lateral view, complementing the inlet and outlet imaging. The resulting anatomical region for selecting the entry point aligns with the recommendations from the literature [29]; however, our findings provide a more extensive assessment regarding implant positioning and deviation of planning in relation to the S1 recess.

Screw mispositioning is a rare but potentially severe complication in transsacral screw osteosynthesis. Iatrogenic injury to neurovascular structures, including the L4 and L5 nerves, internal iliac vessels, S1 nerve, and residual nerve pairs in the spinal canal are a significant concern. Advanced techniques such as CT navigation, 3D modelling, and printing have been shown to help avoid this complication [30]. However, these technologies are not widely accessible or affordable for many healthcare providers and systems. If fluoroscopic visualization of relevant landmarks is refined these feared complications due to their potential impact on patient outcomes may be avoided.

There are several limitations to our study that should be acknowledged. Firstly, the sample size was limited to only 14 cadaveric pelves, and we did not perform a priori power analysis to determine the appropriate sample size. There was no demographic information available. Additionally, we did not use CT scans to assess the transsacral corridors, which have been previously recommended. The Mendel triangle has limitations, dysmorphic sacra can still exhibit a traversable S1 screw corridor, highlighting a discrepancy with the functional definition of pelvic dysmorphism. Furthermore, all pelves used in the study were intact, and it is possible that the lateral projection characteristics described may not be identifiable in cases of displaced fractures. However, we believe that in cases of nondisplaced and minimally displaced fractures, the anatomical landmarks can still be visualized. Lastly, the absence of soft tissues in the cadaveric specimens may have influenced the ease of image evaluation, as soft tissues can often hinder proper visualization during surgery. The clinical application through follow-up studies is still pending. This implies that the proposed safe region has not yet be clinically validated. Further research is needed to confirm its reliability in clinical practice. A backup plan should always be in place with careful planning (e.g., lumbopelvic stabilization) and should be taken into account during patient positioning.

## Conclusion

In this anatomical study using human cadaveric pelves, we demonstrated that the S1 body diagonal bisector (posterosuperior to anteroinferior) is consistently anterior to the S1 recess/foramen in the true lateral fluoroscopic projection. When the anterior boundary of the S1 ala, the iliac cortical density lines, and the height and width of the S1 body are visible on lateral fluoroscopic projections, this single view may be sufficient to determine a safe corridor for transiliac-transsacral screw osteosynthesis at the S1 level. Non-dysmorphic pelves consistently have a zone for potential screw placement anterior to the diagonal, distal to the ICD, and posterior to the anterior cortex. Dysmorphic pelves do not. However, further research is needed to validate these findings by correlating them with corridor sizes on CT imaging and assessing the technical feasibility of screw insertion.
